# Biological effects of a new set 1,2,4-triazolo[1,5-*a*]quinazolines on heart rate and blood pressure

**DOI:** 10.1186/1752-153X-8-3

**Published:** 2014-01-15

**Authors:** Rashad Al-Salahi, Kamal-Eldin El-Tahir, Ibrahim Alswaidan, Nabih Lolak, Mohammed Hamidaddin, Mohamed Marzouk

**Affiliations:** 1 Department of Pharmacetical Chemistry, College of Pharmacy, King Saud University, P. O. Box 2457, Riyadh 11451, Saudi Arabia; 2 Department of Pharmacology, College of Pharmacy, King Saud University, P. O. Box 2457, Riyadh 11451, Saudi Arabia; 3 Department of Pharmaceutical Chemistry, College of Pharmacy, Aleppo University, Aleppo, Syria

**Keywords:** 1,2,4-Triazolo[1,5-*a*]quinazoline, Heart rate, Antihypertensive activity

## Abstract

**Background:**

Several quinazoline and triazole derivatives are reported to possess a wide-range of interesting pharmacological effects. Although various triazoloquinazoline subclasses having been synthesized and studied, the preparation of 1,2,4-triazolo[1,5-*a*]quinazolines as antihypertensive agent is still relatively unexplored. In continuation of our earlier research, we aimed at the synthesis and development of various potent antihypertensive 1,2,4-triazoloquinazoline derivatives.

**Results:**

A new series of 1,2,4-triazolo[1,5-*a*]quinazoline derivatives have been synthesized. Their structures were mainly established by spectroscopic methods of analysis (IR, MS, ^1^H and ^13^C NMR). Their *in vivo* antihypertensive activity was evaluated by tail cuff method using Muromachi Blood Pressure Monitor (Model MK 2000) for rats and mice. Some of the tested compounds were found to exhibit valuable effects in terms of heart rate and blood pressure. According to the biological results, some of tested derivatives have abolished completely the tachycardia of the parent compounds and may be studied and modified as potential adrenoblockers and cardiac stimulant.

**Conclusion:**

New series of fifteen 1,2,4-triazolo[1,5-*a*]quinazolines were synthesized by convenient methodology from four key molecules, whereby their structures were established by advanced spectroscopic analyses. Some lead compounds have abolished completely the tachycardia of the parent compounds, that may be examined as potent adrenoblockers and some other compounds seem to be a cardiac stimulant or may be modified to enhance their hypotensive activity.

## Background

Arterial diseases cause more premature deaths than all other disorders such as cancer and infections combined. High blood pressure has been identified as the most powerful one among the major risk factors for arterial diseases [1]. Now-a-days, several efforts have been made in search of potent anti-hypertensive drugs because hypertension was commonly proved to cause heart failure. Quinazolines and their condensed products are reported to possess several interesting pharmacological effects such as antihypertensive [2], antihistaminic [3,4], analgesic, anti-inflammatory [5,6], anticancer [7], and anti-HIV [8] activities. Prazocin, terazocin and doxazocin as quinazoline derived α-1 blockers, are reputed class of antihypertensive agents. In spite of various triazoloquinazoline systems having been synthesized and studied, the elaborating of 1,2,4-triazolo[1,5-a]quinazoline as antihypertensive agent is still relatively unexplored [1]. Moreover, Some of potent antihypertensive 1,2,4-triazoloquinazoline derivatives were reported [1]. In view of these facts and continuation to our earlier reported triazoloquinazolines researches [1,9-12], we aimed at synthesis of various substituted 1,2,4-triazolo[1,5-*a*]quinazolines and evaluation their biological effects on heart rate and blood pressure.

## Results and discussion

### Synthetic chemistry

**Scheme 1:**
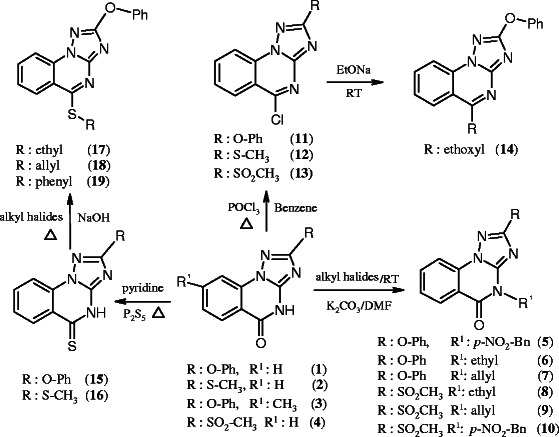
**Synthesis of [1,2,4]triazolo[1,5-*a*]quinazoline derivatives.**

**Figure 1:**
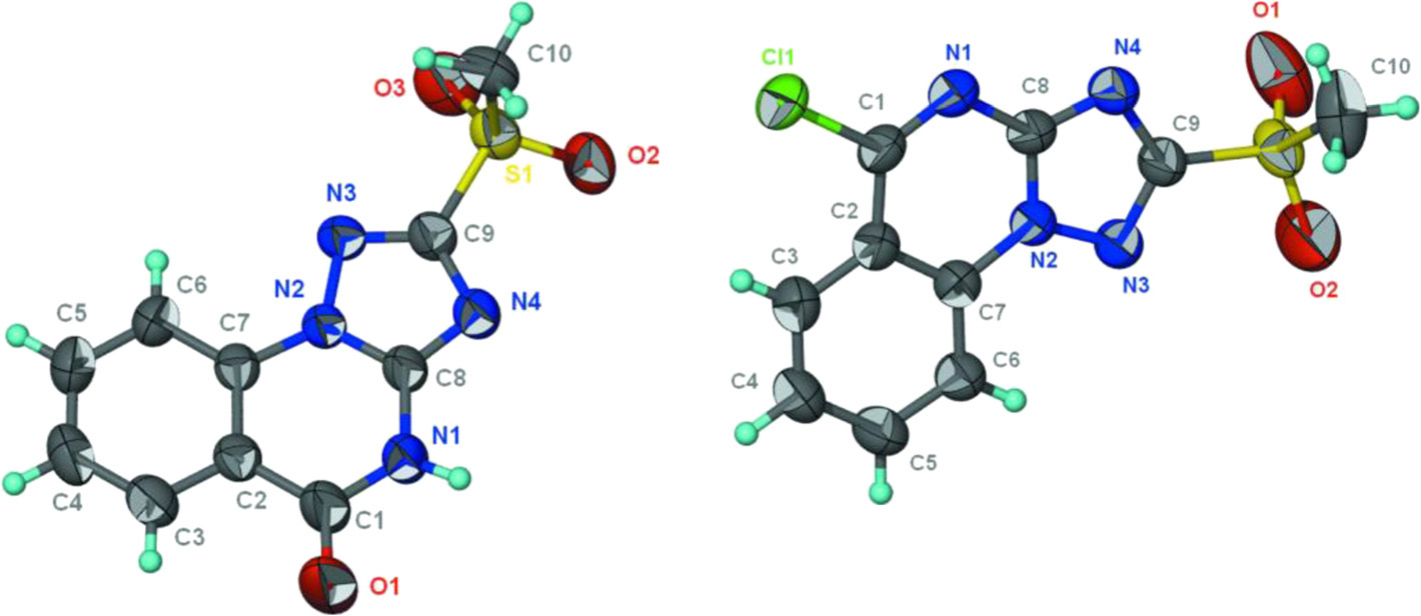
X-ray single crystal 3D-structures of compounds 4 and 13.

As outlined in Scheme 1, the target molecules were synthesized by starting with the preparation of our key materials **(1–4)** according to the literature [13-15]. The structures of **1**–**3** were characterized by NMR, MS, IR spectra and compound **1** has been unambiguously proven by X-ray crystallography [13-18]. Regioselective *N*-alkylation has been well documented in the literature [13,14]. Accordingly, when the triazoloquinazolin-5-ones (**1,4**) were allowed to react with alkyl halides in a molar ratio of 1:1.5 in dry dimethyl formamide at room temperature in the presence of potassium carbonate, the corresponding 4-alkyl[1,2,4]triazolo[1,5-*a*]quinazolin-5-ones (**5**–**10**) resulted in 71-87% yield. The products **5**–**10** were obtained as coloress solid and their IR spectra displayed a strong (C = O) absorption band in the range of 1675–1689 cm^-1^. When equimolar amounts of triazoloquinazolin-5-one **1** and phosphorus pentasulfide were allowed to react in dry pyridine under reflux for 3 h, the desired triazoloquinazolin-5-thione (**15)** could be isolated as yellow solid in excellent yield of 92% [18]. The IR spectra of compound **15** displayed a weak (C = S) absorption band at around 1197 cm^-1^ and characterized by NMR and MS spectra. The structure of compound **16** has been previously proved by NMR, MS and X-ray [14,17]. Treatment of the [1,2,4]triazoloquinazolin-5-thione **15** with different alkyl halides in aqueous sodium hydroxide solution (2 M) afforded smoothly the expected thioethers **(17–19)** in 54-75% yield. Conversion of [1,2,4]triazoloquinazolin-5-ones (**1,2,4**) into 5-chloro-[1,2,4]triazolo[1,5-*a*]quinazolines (**11**–**13**) have been successfully achieved by phosphorus oxychloride in boiling benzene for 2.5 h, followed by treatment with a saturated aqueous solution of potassium carbonate [19]. The formation of **11**–**13** was accompanied by the gradual disappearance of the characteristic (C = O) band of **1** and **2** at 1685–1711 cm^-1^. When compound **11** was reacted at ambient temperature with sodium ethoxide in ethanol, the triazoloquinazoline (**14)** could be obtained in 52% yield. According to literature [20], reaction of compound **2** with hydrogen peroxide in boiling glacial acetic acid, followed by treatment with hot water, the corresponding triazolo[1,5-a]quinazolin-5-one (**4**) was obtained in good yield. IR spectrum of compound **4** is characterized by a strong (C = O) absorption band at 1699 cm^-1^. Analogous to the reaction of **2** with hydrogen peroxide, the corresponding compound **13** was obtained from **12**. The MS spectra of **4** and **13** showed molecular ion peaks at *m/z* 263 and 282 (M^•+^, 100%), corresponding to their molecular formulae. As well as the structures of **4** and **13** were characterized by NMR, MS, IR spectra, and have been unambiguously proven by X-ray crystallography (Figure 1).

Generally, uncorrected melting points of all compounds were recorded and their chemical structures primarily consistent with their IR and MS data (see experimental section) and confirmed by ^1^H- and ^13^C NMR spectroscopy (splitting pattern, *δ*- and *J*-values and comparison with literature of structural related compounds). In the tricyclic nucleus, the benzofused moiety was deduced from its own four one proton ^1^H-signals as two dd (or br d) resonances with *J*_*ortho*_ (7.5-8.5 Hz) and *J*_*meta*_ (1–2 Hz) assignable for H-9 and H-6 and two td (or br t) resonances with *J*_*ortho*_ and *J*_*meta*_ for H-8 and H-7, respectively. The exchangeable NH-proton was interpreted in ^1^H NMR of **3** and **4** at *δ* > 13 ppm and were absent in all spectra of *N*-alkyl or 5-chloro-triazoloquinazolines. ^13^C NMR spectra proved the main tricyclic moiety through characteristic nine resonances including the most downfield key signal of C-2 assigned at ≈ 165 ppm in 2-phenoxy derivatives (*e.g*. **3**, **5**–**7**) that was observed relatively upfield at ≈ 160 in case of 2-methylsulfonyl function (*e.g*. **4**, **8**–**10**) due to the stronger –R and –I (deshielding) effect of *O*-phenoxy than -SO_2_. Another key ^13^C-signal was C-5 that interpreted at *δ* ≈ 158–160 ppm in 5-ones (**3**–**10**), ≈ 167 in 5-Cl derivatives (**11**,**13**) and ≈ 185 in case of 5-thione (**15**). The 2-phenoxy function was easily deduced from its intrinsic three resonances at ≈ 7.46 (td, *J* = 8.5, 2), 7.33 (br d, *J* = 8) and 7.26 (br t, *J* = 7.5) for H-3′/5′, H-2′/6′ and H-4′ (**5**,**10**) and confirmed through its four ^13^C resonances at *δ* ppm ≈ 154, 130, 124 and 119 interpretable for C-1′, 3′/5′, 4′, and 2′/6′, respectively. Methylsulfonyl function was concluded from its own ^1^H and ^13^C resonances of *O*_*2*_*S*-CH_3_ at ≈ 3.45 (s) and 42 ppm, respectively. *N*-alkylation with *p*-nitrobenzyl was unambiguously confirmed by its A_2_M_2_ spin coupling system of the two *ortho*-doublets at about 8.2 and 7.7 (*J* = 8.5) for H-3″/5″ and H-2″/6″ together with the downfield located CH_2_-singlet at ≈ 5.4 and its ^13^C-signal at 46 ppm by the deshielding effect of tertiary *N*-atom (**5**,**10**). Whereas, alkylation with allyl moiety was proved by its four characteristic ^1^H-resonances with intrinsic splitting pattern at about 5.9 (m), 5.25 (dd, *J* = 17.5, 1.5), 5.20 (dd, *J* = 10.5, 1.5) and 4.72 (d, *J* = 5) assignable for olefinic methine and methylene and CH_2_-saturated types (**7**,**9**). The corresponding three ^13^C-resonances were interpreted at *δ* ppm 131.2, 117.5 and 45.2 for C-2, C-3 and C-1 of allyl group, respectively. In compound **18**, 5-*S*-allyl protons were interpreted with the same splitting pattern but highly deshielded at 6.04, 5.42, 5.20 and 4.09 by higher electronegativity of *S*-atom and their ^13^C-signals were reported at 129.6 (C-2″), 115.1 (C-3″), and 32.1 (C-1″). Moreover, *N*-ethyl function was observed in the form of a typical A_2_X_3_ spin coupling system as a quartet (CH_2_) and triplet (CH_3_) at about 4.2 (q, *J* = 7) and 1.3 (t, *J* = 7) and confirmed by their corresponding ^13^C-resonances at *δ* ppm 38.5 and 12.4 (**6**,**8**). In **17** and **19** *S*-ethyl and *S*-phenyl functions showed the same splitting pattern of *N*-ethyl and *O*-phenyl but with slightly different *δ*-values in both ^1^H and ^13^C NMR spectra. All other ^1^H- and ^13^C-resonances of all structures were finally assigned on the basis of comparison with literature data of structure related compounds and according to application of substitution additive rules of ^13^C NMR.

### Antihypertensive activity

*In vivo* antihypertensive activity study of the title compounds **1**–**19** was performed by tail cuff method using Muromachi Blood Pressure Monitor for rats and mice (Model MK 2000). The obtained results, in Table 1, revealed that the nature of substituent and substitution pattern on the tricyclic systems **1**–**4** may have had a considerable impact on the heart rate and blood pressure in all synthesized derivatives (**1**–**19**).

**Table 1:** Effects of compounds 1–19 on heart rate and blood pressure

**Compound**	**% change in heart rate**		**Decrease in the rats arterial pressure (mm H)**
	**Decrease**	**Increase**	
**1**	-	33.3	0
**2**	0	-	0
**3**	-	14.3	6.5
**4**	22.7	-	2.6
**5**	15	-	0
**6**	0	40	0
**7**	5.9	-	0
**8**	14.3	-	0
**9**	20	-	9.1
**10**	5.9	-	0
**11**	0	-	2.6
**12**	16.7	-	2.6 ↑
**13**	-	8.3	0
**14**	-	7.7	7.8
**15**	10	-	5.2
**16**	0	-	0
**17**	5.9	-	6.5
**18**	5.9	-	0
**19**	-	28.5	6.5

↑: Increase.

Basically, the parent **1** was found to increase the heart rate, however it has not demonstrated any effect on blood pressure. Replacement of phenoxy group on position **2** by a sulfanyl group in case of compound **2** has been abolished effect on the heart rate. On the other hand, presence of sulfonyl group in **4** was found to induce the bradycardia, however no effect on blood pressure was noticed. Introduction of methyl group on the lead compound **1** to afford **3** did offer advantageous effect on reduction of blood pressure accomplished by suppressing effect on heart rate in regard to **3**. Alkylation of lactam moiety in **1** furnished *N*-alkylated products **5**–**7**, that did demonstrate remarkable effect. For instance, compound **5** was induced bradycardia, however great increasing in the heart rate showed by **6**, whereas abolished in the tachycardia term and slightly induction on bradycardia was demonstrated by **7**. Similarly, transformation of **4** into **9** has emerged more bradycardia effect accomplished by moderate decreasing on blood pressure. However the product **8** showed induction on bradycardia and **10** exhibited only bradycardia effect. Further transformation of **1** into **11** does not seem to offer any advantages effect on heart rate but a very slightly decrease in blood pressure was observed. Whereas, compound **12** showed more bradycardia and completely abolished in tachycardia effects together with a slightly increase on blood pressure. Moreover, decreasing on tachycardia profile was exhibited by compound **13**. The replacement of the chlorine atom in **11** by ethoxide group gave **14**, which has shown decrease on blood pressure and tachycardia effects. Thionation of **1** into **15** was accomplished by abolishing the tachycardia profile and induce on bradycardia term. The same behavior on tachycardia effect was observed by compound **16** but no influence on blood pressure profile has been recorded. Moreover, the transformation of **15** into **17** demonstrated almost the same behavior in the terms of the effects on heart rate and blood pressure**.** Whereas, compound **18** was exerted complete abolishing in tachycardia and slight induction on bradycardia profiles. However, induction on tachycardia and decreasing on blood pressure terms was demonstrated by compound **19.**

Structure modifications on the lead compounds **1**–**4** have afforded derivatives with different effects on heart rate and blood pressure profiles. Variation in the substituted alkyl groups has demonstrated slightly remarkable activity in regards to the parents, such as increasing on heart rate emerged by **6**, induction in bradycardia proved by **5** and slightly decreasing in blood pressure occurred with **9.** This could be attributed to the characteristic features of their alkyl groups. Introduction of lipophilic group on position 5 of the parent was appeared to offer slightly decrease in blood pressure in case of compounds **11** and **12.** Nevertheless, the slight effect on blood pressure of **11** and **12** bearing at the 5-position a chlorine atom indicates that, not only chloro lipophilic factor, but also the sterric effect are important for decrease on blood pressure as shown by **14**. Thionation targets **15** and **16** showed remarkable attenuated on profiles activity, despite these compounds possess enhanced lipophilicity comparable to that parent compounds. However thioether products have almost emerged the same behavior on blood pressure and heart rate as in **17**, and decreasing on blood pressure by **19.** The actual explanation for these changes regarding SAR will await the elucidation of the mechanism(s) of action of the compounds.

### Experimental

#### ***General***

Melting points were determined on open glass capillaries using a Mettler FP 62 apparatus and are uncorrected. The IR (KBr, ѵ, cm^-1^) spectra were recorded on a Perkin Elmer FT-IR Spectrum BX system. NMR spectra were recorded on a Bruker AMX 500 spectrometer in DMSO-*d*_*6*_ and reported as *δ* ppm values relative to TMS at 500 and 125 MHz for ^1^H- and ^13^C NMR, respectively. Mass spectra were measured on an Agilent 6410 TSQ system connected to Agilent 1200 HPLC interface (samples were infused in MeOH). Follow up of the reactions and checking the purity of compounds was made by TLC on DC-Mikrokarten polygram SIL G/UV254, from the Macherey-Nagel Firm, Duren Thickness: 0.25 mm. Column chromatography was conducted on silica gel (ICN Silica 100–200, active 60 Å).

#### ***8-Methyl-2-phenoxy-4H-[1,2,4]triazolo[1,5-a]quinazolin-5-one (3)***

2-Hydrazino-5-methyl benzoic acid (10 mmol) was added portion wise to a stirred solution of diphenyl-*N-*cyanoimidocarabonate (10 mmol) in EtOH (20 mL) at 0°C. Afterwards, triethylamine (30 mmol) was added drop-wise over a period of 30 min. After the addition was complete, the reaction mixture was left to stirr overnight at room temperature. Acidification of the mixture was performed by conc. HCl under ice cooling followed by refluxing for 1–3 h. After cooling, the mixture was poured into ice/water, the resulting solid was filtered, washed with water and dried. Recrystallization from THF gave analytically pure colored as white solid, yield: 77%, m.p. 218–220°C; IR (KBr): *v*/cm^-1^ 1692, (C = O), 3219 (NH); ^1^H NMR (DMSO-*d*_*6*_): *δ* = 13.04 (br s, 1H, NH), 7.98 (br s, 1H, H-6), 7.72 (br s, 2H, H-7,9), 7.45 (td, *J* = 8.5, 2 Hz, 2H, H-3′/5′), 7.32 (br d, *J* = 8 Hz, 2H, H-2′/6′), 7.25 (br t, *J* = 7.5 Hz, 1H, H-4′), 2.45 (s, 3H, Ar-CH_3_); ^13^C NMR (DMSO-*d*_*6*_): *δ* = 165.7 (C-2), 159.4 (C-5), 154.2 (C-1′), 147.1 (C-9a), 136.2 (C-8), 135.3 (C-3a), 133.6 (C-5a), 129.7 (C-3′/5′), 127.8 (C-6), 124.9 (C-4′), 119.4 (C-2′/6′), 116.6 (C-7), 114.1 (C-9), 20.5 (Ar-CH_3_); MS (ESI) negative mode: *m/z* 291.1 (M^-^ - 1) for MW = 292.

#### ***2-Methylsulfonyl-4H-[1,2,4]triazolo[1,5-a]quinazolin-5-one (4)***

An amount of 2-methylsulfanyl-4H-[1,2,4]triazolo[1,5-a]quinazolin-5-one (1 mmol) was dissolved in bioling glacial acetic acid (10 mL), afterward H_2_O_2_ (10 mL), was added drop-wise over a period of 5 min. while heating. After the addition was complete, the mixture was poured into hot water and left at room temperature, the resulting solid was filtered, washed with water and dried. Recrystallization from toluene gave analytically pure colored as white solid, yield: 50%, m.p. 261–263°C; IR (KBr): *v*/cm^-1^ 1690 (C = O) 3240 (NH); ^1^H NMR (DMSO-*d*_*6*_): *δ* = 13.39 (br s, 1H, NH), 8.22 (br d, *J* = 8 Hz, 1H, H-9), 8.04 (br d, *J* = 8 Hz, 1H, H-6), 7.97 (br t, *J* = 7.5 Hz, 1H, H-8), 7.65 (br t, *J* = 7.5 Hz, 1H, H-7), 3.35 (s, 3H, *O*_*2*_*S*-CH_3_); ^13^C NMR (DMSO-*d*_*6*_): *δ* = 160.9 (C-2), 160.2 (C-5), 149.9 (C-9a), 135.4 (C-8), 135.3 (C-3a), 128.4 (C-6), 127.2 (C-5a), 118.0 (C-7), 114.8 (C-9), 42.0 (*O*_*2*_*S*-CH_3_); MS (ESI) (negative mode): *m/z* 263.1 (M^-^ - 1), (positive mode): *m/z* 265.0 (M^+^ + H), 287.1 (M^+^ + Na) for MW = 264.

#### ***General procedure for synthesis of 2-phenoxy(methylsulfonyl)-4-alkyl(aralkyl)-[1,2,4]-triazolo[1,5-a]quinazolin-5-ones (5–10)***

To a solution of **1** or **4** (I mmol) in DMF (5 mL) was added potassium carbonate (1.2 mmol) portion wise over a period of 10 min at room temperature. After stirring for 20 min, the appropriate alkyl halide (1.5 mmol) was added drop wise, and the reaction mixture was stirred for 18 h at room temperature. The mixture was poured into ice/water, the precipitate was filtered off, washed with water and dried. Analytically pure products **5**–**10** were obtained after recrystallization from THF.

#### ***2-Phenoxy-4-(p-nitrobenzyl)-[1,2,4]triazolo[1,5-a]quinazolin-5-one (5)***

White solid, yield: 78%, m.p. 190–192°C; IR (KBr): *v*/cm^-1^ 1677 (C = O); ^1^H NMR (DMSO-*d*_*6*_): *δ* = 8.25 (dd, *J* = 8, 1 Hz, 1H, H-9), 8.19 (d, *J* = 8.5 Hz, 2H, H-3″/5″), 7.95 (td, *J* = 8.5, 1.5 Hz, 1H, H-8), 7.87 (br d, *J* = 8 Hz, 1H, H-6), 7.71 (d, *J* = 8.5 Hz, 2H, H-2″/6″), 7.59 (td, *J* = 8, 1 Hz, 1H, H-7), 7.46 (td, *J* = 8.5, 2 Hz, 2H, H-3′/5′), 7.33 (br d, *J* = 8 Hz, 2H, H-2′/6′), 7.26 (br t, *J* = 7.5 Hz, 1H, H-4′), 5.43 (s, 2H, –CH_2_-Ar); ^13^C NMR (DMSO-*d*_*6*_): *δ* = 165.2 (C-2), 158.7 (C-5), 154.1 (C-1′), 148.4 (C-9a), 146.9 (C-4″), 143.4 (C-1″), 135.8 (C-8), 135.3 (C-3a), 129.8 (C-3′/5′), 128.8 (C-2″/6″), 128.6 (C-6), 126.0 (C-5a), 124.9 (C-4′), 121.5 (C-3″/5″), 119.0 (C-2′/6′), 116.2 (C-7), 114.2 (C-9), 46.1 (-CH_2_-Ar); MS (EI): *m/z* (%) 413 (M^•+^, 92) for MW = 413.

#### ***4-Ethyl-2-phenoxy-[1,2,4]triazolo[1,5-a]quinazolin-5-one (6)***

White solid, yield: 73%, m.p. 165–167°C; IR (KBr): *v*/cm^-1^ 1675 (C = O ); ^1^H NMR (DMSO-*d*_*6*_): *δ* = 8.23 (dd, *J* = 8, 1 Hz, 1H, H-9), 7.92 (td, *J* = 8.5, 1.5 Hz, 1H, H-8), 7.83 (br d, *J* = 8 Hz, 1H, H-6), 7.56 (td, *J* = 8.5, 1 Hz, 1H, H-7), 7.46 (td, *J* = 8.5, 1 Hz, 2H, H-3′/5′), 7.35 (dd, *J* = 8.5, 1 Hz, 2H, H-2′/6′), 7.27 (br t, *J* = 7.5 Hz, 1H, H-4′), 4.15 (q, *J* = 7 Hz, 2H, –CH_2_-CH_3_), 1.29 (t, *J* = 7 Hz, 3H, –CH_2_-CH_3_); ^13^C NMR (DMSO-*d*_*6*_): *δ* = 165.4 (C-2), 158.3 (C-5), 154.2 (C-1′), 148.2 (C-9a), 135.4 (C-8), 135.0 (C-3a), 129.8 (C-3′/5′), 128.4 (C-6), 125.8 (C-5a), 124.9 (C-4′), 119.1 (C-2′/6′), 116.3 (C-7), 114.1 (C-9), 38.5 (-CH_2_-CH_3_), 12.4 (-CH_2_-CH_3_); MS (EI): *m/z* (%) 306 (M^•+^, 98) for MW = 306.

#### ***4-Allyl-2-phenoxy-[1,2,4]triazolo[1,5-a]quinazolin-5-one (7)***

White solid, yield: 85%, m.p. 133–135°C; IR (KBr): *v*/cm^-1^ 1684 (C = O); ^1^H NMR (DMSO-*d*_*6*_): *δ* = 8.23 (br d, *J* = 8 Hz, 1H, H-9), 7.92 (td, *J* = 8.5, 1.5 Hz, 1H, H-8), 7.83 (br d, *J* = 8 Hz, 1H, H-6), 7.57 (td, *J* = 8.5, 1 Hz, 1H, H-7), 7.47 (td, *J* = 8.5, 1 Hz, 2H, H-3′/5′), 7.35 (dd, *J* = 8.5, 1 Hz, 2H, H-2′/6′), 7.27 (br t, *J* = 7.5 Hz, 1H, H-4′), 5.97 (m, 1H, H-2″), 5.24 (dd, *J* = 17.5, 1.5 Hz, 1H, H-3a″), 5.19 (dd, *J* = 10.5, 1.5 Hz, 1H, H-3b″), 4.72 (d, *J* = 5 Hz, 2H, H-1″); ^13^C NMR (DMSO-*d*_*6*_): *δ* = 165.4 (C-2), 158.2 (C-5), 154.2 (C-1′), 148.2 (C-9a), 135.5 (C-8), 135.1 (C-3a), 131.2 (C-2″), 129.8 (C-3′/5′), 128.5 (C-6), 125.9 (C-5a), 124.9 (C-4′), 119.1 (C-2′/6′), 117.5 (C-3″), 116.1 (C-7), 114.1 (C-9), 45.2 (C-1″); MS (EI): *m/z* (%) 318 (M^•+^, 98) for MW = 318.

#### ***4-Ethyl-2-methylsulfonyl-[1,2,4]triazolo[1,5-a]quinazolin-5-one (8)***

White solid, yield: 71%, m.p. 187–189°C; IR (KBr): *v*/cm^-1^ 1675 (C = O); ^1^H NMR (DMSO-*d*_*6*_): *δ* = 8.27 (br d, *J* = 8 Hz, 1H, H-9), 8.07 (br d, *J* = 8 Hz, 1H, H-6), 8.00 (br t, *J* = 7.5 Hz, 1H, H-8), 7.69 (br t, *J* = 7.5 Hz, 1H, H-7), 4.22 (q, *J* = 6.5 Hz, 2H, –CH_2_–CH_3_), 3.49 (s, 3H, *O*_*2*_*S* -CH_3_), 1.34 (t, *J* = 6.5 Hz, 3H, –CH_2_–CH_3_); ^13^C NMR (DMSO-*d*_*6*_): *δ* = 160.8 (C-2), 158.3 (C-5), 149.8 (C-9a), 135.5 (C-8), 134.5 (C-3a), 128.6 (C-6), 127.6 (C-5a), 117.4 (C-7), 114.8 (C-9), 42.1 (*O*_*2*_*S* -CH_3_), 38.9 (-CH_2_-CH_3_), 12.4 (-CH_2_–CH_3_); MS (EI): *m/z* (%) 292 (M^•+^, 89 ) for MW = 292.

#### ***4-Allyl-2-methylsulfonyl-[1,2,4]triazolo[1,5-a]quinazolin-5-one (9)***

White solid, yield: 87%, m.p. 152–154°C; IR (KBr): *v*/cm^-1^ 1684 (C = O); ^1^H NMR (DMSO-*d*_*6*_): *δ* = 8.27 (br d, *J* = 8 Hz, 1H, H-9), 8.10 (br d, *J* = 8 Hz, 1H, H-6), 8.03 (br t, *J* = 7.5 Hz, 1H, H-8), 7.70 (br t, *J* = 7.5 Hz, 1H, H-7), 6.00 (m, 1H, H-2′), 5.31 (br d, *J* = 17.5 Hz, 1H, H-3a′), 5.21 (br d, *J* = 10.5 Hz, 1H, H-3b′), 4.80 (d, *J* = 5 Hz, 2H, H-1′), 3.48 (s, 3H, *O*_*2*_*S* –CH_3_); ^13^C NMR (DMSO-*d*_*6*_): *δ* = 160.7 (C-2), 158.3 (C-5), 149.9 (C-9a), 135.7 (C-8), 134.8 (C-3a), 131.0 (C-2′), 128.7 (C-6), 127.7 (C-5a), 117.6 (C-3′), 117.2 (C-7), 114.9 (C-9), 45.5 (C-1′), 42.0 (*O*_*2*_*S* -CH_3_); MS (EI): *m/z* (%) 304 (M^•+^, 100) for MW = 304.

#### ***4-(p-Nitrobenzyl)-2-methylsulfonyl-[1,2,4]triazolo[1,5-a]quinazolin-5-one (10)***

White solid, yield: 72%, m.p. 199–201°C; IR (KBr): *v*/cm^-1^ 1678 (C = O); ^1^H NMR (DMSO-*d*_*6*_): *δ* = 8.29 (br d, *J* = 8 Hz, 1H, H-9), 8.19 (d, *J* = 8.5 Hz, 2H, H-3′/5′), 8.14 (br d, *J* = 8 Hz, 1H, H-6), 8.05 (br t, *J* = 7.5 Hz, 1H, H-8), 7.75 (d, *J* = 8.5 Hz, 2H, H-2″/6″), 7.72 (br t, *J* = 7.5 Hz, 1H, H-7), 5.51 (s, 2H, –CH_2_-Ar), 3.47 (s, 3H, *O*_*2*_*S* –CH_3_); ^13^C NMR (DMSO-*d*_*6*_): *δ* = 160.6 (C-2), 158.8 (C-5), 150.2 (C-9a), 146.9 (C-4′), 143.2 (C-1′), 135.9 (C-8), 135.0 (C-3a), 128.7 (C-2′/6′), 128.7 (C-6), 127.7 (C-5a), 123.5 (C-3′/5′), 117.3 (C-7), 114.9 (C-9), 46.5 (-CH_2_-Ar), 42.0 (*O*_*2*_*S* –CH_3_); MS (EI): *m/z* (%) 399 (M^•+^,90 ) for MW = 399.

#### ***5-Chloro-2-phenoxy-[1,2,4]triazolo[1,5-a]quinazoline (11)***

Compound **1** (1 mmol) was refluxed with Phosphorous oxychloride (1 mL) in benzene (7 mL) for 2.5 h. The solvent was evaporated and the residue was treated with saturated solution of potassium carbonate. The solid was filtered, washed thoroughly with water, dried and recrystallized from THF to give pure compound as white solid, yield: 82%, m.p. 167–169°C; ^1^H NMR (DMSO-*d*_*6*_): *δ* = 8.07 (br d, *J* = 8 Hz, 1H, H-9), 7.64 (m, 2H, H-6/8), 7.40 (td, *J* = 8.5, 1 Hz, 2H, H-3′/5′), 7.30 (td, *J* = 8.5, 1 Hz, 1H, H-7), 7.25 (dd, *J* = 8.5, 1 Hz, 2H, H-2′/6′), 7.18 (br t, *J* = 8 Hz, 1H, H-4′); ^13^C NMR (DMSO-*d*_*6*_): *δ* = 167.6 (C-2), 166.3 (C-5), 157.0 (C-9a), 155.0 (C-1′), 136.3 (C-3a), 131.7 (C-8), 129.4 (C-3′/5′), 128.0 (C-7), 123.8 (C-4′), 123.0 (C-6), 119.0 (C-2′/6′), 118.6 (C-5a), 112.9 (C-9). MS (EI): *m/z* (%) 296 (M^•+^, 100) for MW = 296.

#### ***5-Chloro-2-methylsulfonyl-[1,2,4]triazolo[1,5-a]quinazoline (13)***

1 mmol of 2-methylsulfanyl-5-chloro-[1,2,4]triazolo[1,5-*a*]quinazoline was dissolved in bioling glacial acetic acid (10 mL), afterward H_2_O_2_ (10 mL) was added drop-wise over a period of 5 min. while heating. After the addition was complete, the mixture was poured into hot water and lef at room temperature, the resulting solid was filtered, washed with water and dried. Recrystallization from Toluene gave analytically pure colored as white solid, yield: 62%, m.p. 196–198°C; ^1^H NMR (DMSO-*d*_*6*_): *δ* = 8.21 (br d, *J* = 8 Hz, 1H, H-9), 7.99 (m, 2H, H-6/8), 7.66 (br t, *J* = 7 Hz, 1H, H-7), 3.54 (s, 3H, *O*_*2*_*S*-CH_3_); ^13^C NMR (DMSO-*d*_*6*_): *δ* = 162.9 (C-5), 160.9 (C-2), 158.6 (C-9a), 137.3 (C-3a), 135.5 (C-8), 128.8 (C-7), 127.4 (C-6), 118.5 (C-5a), 114.7 (C-9), 42.1 (*O*_*2*_*S*-CH_3_); MS (EI): *m/z* (%) 282 (M^•+^, 100) for MW = 282.

#### ***5-Ethoxy-2-phenoxy-[1,2,4]triazolo[1,5-a]quinazoline (14)***

A freshly prepared sodium ethoxide solution from sodium (150 mg) and absolut ethanol (35 mL) was reacted with compound **11** (1 mmol) by stirring at room temperature for 30 min. Afterwards the solid was collected by filtration, air dried, and recrystallized from THF to give product as white solid, yield: 56%, m.p. 197–199°C; IR (KBr): *v*/cm^-1^ 1602 (C = N); ^1^H NMR (DMSO-*d*_*6*_): *δ* = 8.15 (br d, *J* = 8 Hz, 1H, H-9), 7.71 (br t, *J* = 7.5 Hz, 1H, H-8), 7.65 (br d, *J* = 8 Hz, 1H, H-6), 7.49 (dt, *J* = 8.5, 1 Hz, 2H, H-3′/5′), 7.43 (br t, *J* = 7.5 Hz, 1H, H-7), 7.36 (dd, *J* = 8.5, 1 Hz, 2H, H-2′/6′), 7.04 (br t, *J* = 8 Hz, 1H, H-4′), 3.72 (q, *J* = 7 Hz, 2H, CH_2_), 1.57 (t, *J* = 7 Hz, 3H, CH_3_); ^13^C NMR (DMSO-*d*_*6*_): *δ* = 167.3 (C-2), 155.5, (C-5), 154.2 (C-9a), 150.7 (C-1′), 135.6 (C-3a), 133.5 (C-8), 129.8 (C-3′/5′), 127.9 (C-7), 123.8 (C-4′), 123.0 (C-6), 119.6 (C-2′/6′), 118.9 (C-5a), 112.9 (C-9), 24.1 (-CH_2_CH_3_), 13.9 (-CH_2_CH_3_); MS (EI): *m/z* (%) 306 (M^•+^, 73) for MW = 306.

#### ***2-Phenoxy-4H-[1,2,4]triazolo[1,5-a]quinazolin-5-thione (15)***

Compounds **1** (1 mmol) was refluxed with phosphorous pentasulfide (1 mmol) in absolute pyridine (5 mL) for 3 h. Afterwards the reaction mixture was cooled and poured into ice/water, the yellow precipitate was separated by filtration and washed thoroughly with water. Recrystallization from aqueous dimethylformamide furnished analytically pure as yellow solid, yield: 92%, m.p. 209–211°C; IR (KBr): *v*/cm^-1^ 1197 (C = S); ^1^H NMR (DMSO-*d*_*6*_): *δ* = 8.64 (dd, *J* = 8, 1 Hz, 1H, H-9), 7.95 (br dt, *J* = 7.5, 1 Hz, 1H, H-8), 7.89 (br d, *J* = 7.5 Hz, 1H, H-6), 7.56 (td, *J* = 8, 1 Hz, 1H, H-7), 7.47 (td, *J* = 8.5, 1 Hz, 2H, H-3′/5′), 7.35 (dd, *J* = 8.5, 1 Hz, 2H, H-2′/6′), 7.27 (br t, *J* = 7.5 Hz, 1H, H-4′); ^13^C NMR (DMSO-*d*_*6*_): *δ* = 185.3 (C-5), 165.0 (C-2), 154.2 (C-1′), 149.3 (C-9a), 135.9 (C-8),132.4 (C-3a), 131.8 (C-5a), 129.8 (C-3′/5′), 126.3 (C-6), 125.0 (C-4′), 123.9 (C-7), 119.4 (C-2′/6′), 114.5 (C-9); MS (EI): *m/z* (%) 294 (M^+^, 90) for MW = 294.

#### ***General procedure for synthesis of 5-Alkyl(phenyl)sulfanyl-2-phenxy-[1,2,4]triazolo[1,5-a]-quinazolines (17–19)***

Compound **15** (1 mmol) was dissolved in 2 M sodium hydroxide solution (10 mL), alkyl halide (1.5 mmol) was added dropwise over a period 2 min, the mixture was left to stir for I h at room temperature, and the obtained solid was separated by filtration, washed thoroughly with water and dried. Recrystallization of the crude products from THF afforded **17**–**19** as colored pure solid.

#### ***5-Ethylsulfanyl-2-phenoxy-[1,2,4]triazolo[1,5-a]quinazoline (17)***

White solid, yield: 68%, m.p. 185–187°C; ^1^H NMR (DMSO-*d*_*6*_): *δ* = 8.26 (br d, *J* = 8 Hz, 1H, H-9), 7.75 (br t, *J* = 7.5 Hz, 1H, H-8), 7.77 (br d, *J* = 8 Hz, 1H, H-6), 7.63 (dt, *J* = 8.5, 1 Hz, 2H, H-3′/5′), 7.51 (br t, *J* = 7.5 Hz, 1H, H-7), 7.41 (dd, *J* = 8.5, 1 Hz, 2H, H-2′/6′), 7.11 (br t, *J* = 8 Hz, 1H, H-4′), 4.39 (q, *J* = 7.2 Hz, 2H, CH_2_), 1.41 (t, *J* = 7.2 Hz, 3H, CH_3_); ^13^C NMR (DMSO-*d*_*6*_): *δ* = 167.9 (C-2), 156.7, (C-5), 154.0 (C-9a), 150.9 (C-1′), 136.1 (C-3a),134.8 (C-8), 130.1 (C-3′/5′), 128.3 (C-7), 125.2 (C-4′), 123.9 (C-6), 120.3 (C-2′/6′), 119.1 (C-5a), 112.1 (C-9), 62.1 (-CH_2_CH_3_), 13.9 (-CH_2_CH_3_); MS (EI): *m/z* (%) 322 (M^•+^, 72) for MW = 322.

#### ***5-Allylsulfanyl-2-phenoxy-[1,2,4]triazolo[1,5-a]quinazoline (18)***

Yellow solid, yield: 75%, m.p. 109–111°C; ^1^H NMR (DMSO-*d*_*6*_): *δ* = 8.08 (br d, *J* = 8 Hz, 1H, H-9), 7.72 (td, *J* = 8.5, 1.5 Hz, 1H, H-8), 7.66 (br d, *J* = 8 Hz, 1H, H-6), 7.37 (td, *J* = 8.5, 1 Hz, 2H, H-3′/5′), 7.33 (td, *J* = 8.5, 1 Hz, 1H, H-7), 7.25 (dd, *J* = 8.5, 1 Hz, 2H, H-2′/6′), 7.17 (br t, *J* = 7.5 Hz, 1H, H-4′), 6.04 (m, 1H, H-2″), 5.42 (dd, *J* = 17.5, 1.5 Hz, 1H, H-3a″), 5.20 (dd, *J* = 10.5, 1.5 Hz, 1H, H-3b″), 4.09 (d, *J* = 6.5 Hz, 2H, H-1″); ^13^C NMR (DMSO-*d*_*6*_): *δ* = 167.4 (C-2), 166.6 (C-5), 154.9 (C-1′), 154.2 (C-5a), 151.0 (C-9a), 136.2 (C-3a), 135.7 (C-8), 129.6 (C-2″), 129.5 (C-3′/5′), 127.9 (C-6), 125.0 (C-7), 123.9 (C-4′), 119.0 (C-2′/6′), 115.1 (C-3″), 112.9 (C-9), 32.1 (C-1″); MS (EI): *m/z* (%) 334 (M^•+^, 89) for MW = 334**.**

#### ***2-Phenoxy-5-phenylsulfanyl-[1,2,4]triazolo[1,5-a]quinazoline (19)***

Yellow solid, yield: 54%, m.p. 172–174°C; ^1^H NMR (DMSO-*d*_*6*_): *δ* = 8.81 (br dd, *J* = 8.5, 1 Hz, 1H, H-9), 7.78 (br d, *J* = 8 Hz, 1H, H-6), 7.71 (td, *J* = 8.5, 1.5 Hz, 1H, H-8), 7.66 (td, *J* = 8.5, 1 Hz, 1H, H-7), 7.43 (td, *J* = 8.5, 1 Hz, 2H, H-3′/5′), 7.38(td, *J* = 8.5, 1 Hz, 2H, H-3″/5″), 7.31 (dd, *J* = 8.5, 1 Hz, 2H, H-2′/6′), 7.27 (dd, *J* = 8.5, 1 Hz, 2H, H-2″/6″), 7.21 (br t, *J* = 8 Hz, 1H, H-4′), 7.17 (br t, *J* = 7.5 Hz, 1H, H-4″); ^13^C NMR (DMSO-*d*_*6*_): *δ* = 166.8 (C-2), 155.0 (C-5), 154.8 (C-1′), 152.4 (C-3a), 132.9 (C-9a), 132.3 (C-8), 132.1 (C-3″/5″), 129.5 (C-3′/5′, 1″), 129.4 (C-4″), 127.9 (C-5a), 124.2 (C-4′,2″/6″), 123.8 (C-6), (C-3′/5′), 119.1 (C-2′/6′), 119.0 (C-7), 113.5 (C-9); MS (EI): *m/z* (%) 370 (M^+^, 74 ) for MW = 370.

#### ***Antihypertensive activity***

*In vivo* antihypertensive activity study of the title compounds was performed by tail cuff method using Blood Pressure Monitor for rats and mice (Model MK 2000- Muromachi kikkai Co. ltd. Japan). The samples of the investigated compounds were administered orally at a dose of 5 mg/kg as suspension in 1% sodium carboxy methyl cellulose. Measurements (blood pressure and heart rate) were recorded before and after the treatment of tested compounds at the intervals of 1 h for 5 h.

## Conclusions

Since compounds **4**, **8**, **9**, **12**, and **15** have abolished completely the tachycardia of the parent compounds, they may be studied as potential adrenoblockers. Compounds **9** and **14** may be modified to enhance their hypotensive activity. Furthermore, compound **6** seem to be a cardiac stimulant and it will be studied further for this concern. Finally, the structure–activity relationship (SAR) study of the compounds gave us some useful insights about the characteristic requirements, which may be taken into consideration in the design of new antihypertensive agents.

## Competing interests

The authors declare that they have no competing interests.

## Authors’ contributions

RA and NL have made a substantial contribution to experimental design. RA and MM made a significant contribution to acquisition of data, analysis, manuscript preparation. KE designed and performed the biological study and manuscript revision. IA and MH read, revised and approved the final manuscript. All authors read and approved the final manuscript.
